# The burden of chronic kidney disease attributed to metabolic risk factors: Insights from the 2021 global burden of disease study

**DOI:** 10.12669/pjms.42.7.13787

**Published:** 2026-07

**Authors:** Yunteng Yao, Quying Qiu, Xiaohua Zhang, Ping Li, Lei Zhou

**Affiliations:** 1 Yunteng Yao, Internal Medicine, Qingpu Branch of Zhongshan Hospital, Fudan University, Shanghai 201700, Shanghai, China; 2 Quying Qiu, Internal Medicine, Qingpu Branch of Zhongshan Hospital, Fudan University, Shanghai 201700, Shanghai, China; 3 Xiaohua Zhang Department of Nephrology, Qingpu Branch of Zhongshan Hospital, Fudan University, Shanghai 201700, Shanghai, China; 4 Ping Li Surgical Intensive Care Unit, Tongren Hospital Affiliated to Shanghai Jiao Tong University, Shanghai 200336, Shanghai, China; 5 Lei Zhou Department of Pulmonary and Critical Care Medicine, Qingpu Branch of Zhongshan Hospital, Fudan University, Shanghai 201700, Shanghai, China. Shanghai Key Laboratory of Medical Internet of Things, Shanghai 201700, Shanghai, China

**Keywords:** Chronic kidney disease, Metabolic risk, Global burden of disease, Trend projections

## Abstract

**Objective:**

To assess the burden and trends of chronic kidney disease(CKD) attributable to metabolic risk factors from 1990 to 2021 and to project its burden to 2040 using Global Burden of Disease Study 2021 data.

**Methodology:**

This retrospective descriptive epidemiological study used publicly available GBD 2021 data for 204 countries and territories, downloaded on June 30, 2025. Deaths, disability-adjusted life years(DALYs), age-standardized mortality rate(ASMR), and age-standardized DALY rate (ASDR) were analyzed by sex, age, Socio-demographic Index(SDI), region, and country. Trends were assessed using estimated annual percentage change, and projections for 2022–2040 were generated using a Bayesian age-period-cohort model.

**Results:**

In 2021, the global ASMR and ASDR were 18.49 and 529.43 per 100,000 population, respectively. From 1990 to 2021, deaths increased from 552,433.01 to 1,527,039.62, and DALYs rose from 20,731,495.66 to 44,438,400.64. The burden was higher in males, increased with age, and varied markedly across SDI regions. Low-SDI regions had the highest ASMR and ASDR, whereas high-SDI regions showed the fastest increase over time. High fasting plasma glucose was the leading contributor, followed by high systolic blood pressure and high body mass index. ASMR is projected to continue rising slowly to 2040, while ASDR may decline slightly.

**Conclusion:**

The CKD attributable to metabolic risk factors remains a growing and unevenly distributed global burden. Prevention should be SDI-stratified, emphasizing obesity control in high-SDI regions, early screening and basic management of diabetes and hypertension in low-SDI regions, and guideline-based kidney-protective therapies, including sodium-glucose cotransporter-2 inhibitors for eligible patients.

## INTRODUCTION

Chronic kidney disease(CKD), defined by renal impairment or sustained glomerular filtration rate (GFR) <60 mL/min/1.73 m² for ≥3 months, is a major global health issue.^1^,^2^ In 2021, CKD affected 697 million people and accounted for 44.5 million disability-adjusted life years (DALYs), ranking 11th among global burdens.^3^,^4^ CKD leads to complications such as cardiovascular disease, anemia, and neurological disorders, reducing quality of life and increasing mortality.^5^ Among modifiable risk factors, metabolic abnormalities are key drivers of CKD onset and progression.^6^

The Global Burden of Disease (GBD) 2021 dataset provides stratified estimates of disease burden and attributable risk by demographic and geographic variables.^7^ In the GBD risk hierarchy, metabolic risk factors refer to physiological exposures. For CKD, the present analysis focused on high fasting plasma glucose (FPG), high systolic blood pressure (SBP), and high body mass index (BMI); impaired kidney function was not treated as an upstream exposure for CKD attribution. While most studies have focused on single diseases or isolated risks,^8^ comprehensive analyses of CKD attributable to these metabolic determinants remain limited.

Using GBD 2021, this study evaluates the CKD burden attributable to metabolic risk factors from 1990 to 2021, examines disparities by age, sex, Socio-demographic Index (SDI), region, and country, and projects trends through 2040. The findings provide evidence for SDI- and risk-specific prevention strategies in kidney health.

## METHODOLOGY

This retrospective descriptive epidemiological study was conducted at the Qingpu Branch of Zhongshan Hospital, Fudan University, Shanghai, China and analyzed the global burden of metabolic-risk-attributable CKD from 1990 to 2021 using publicly available GBD 2021 estimates. The study period was defined by the GBD 2021 time series, and analyses were completed in June 2025. In this analysis, metabolic risk factors were restricted to high fasting plasma glucose, high systolic blood pressure, and high body mass index; impaired kidney function was not treated as an upstream exposure for CKD attribution. Estimates were stratified by sex, age, Socio-Demographic Index (SDI), GBD region and country; a Bayesian age-period-cohort (BAPC) model was used to project the burden for 2022-2040.

### Ethical considerations:

Since all the data used in the study is available in public domain, and there was no intervention, there was no need for an ethical approval.

Information on CKD related to metabolic risk factors was retrieved from the Global Burden of Disease 2021 database (http://ghdx.healthdata.org/gbd-results-tool). Data were downloaded on June 30, 2025 after the public release of the GBD 2021 estimates, and the selected database version was checked against the GBD 2021 disease and risk-factor hierarchy before analysis. The dataset comprised major indicators, including deaths, disability-adjusted life years (DALYs), years of life lost (YLL), years lived with disability (YLD), the age-standardized mortality rate (ASMR), and the age-standardized DALY rate (ASDR). Because only aggregated, de-identified and publicly available data were used, additional ethics committee/IRB approval was not required.

Per ICD-10, CKD is defined as sustained structural or functional kidney impairment lasting >3 months (ICD-10: N18).^4^ The SDI combines per-capita income, mean years of schooling and total fertility to reflect regional development; GBD 2021 groups the world into 21 epidemiologically similar regions and stratifies countries into five SDI quintiles.^9^ To enable comparability across populations, age-standardized rates (ASRs) were used.^10^ DALYs = YLL + YLD, and YLL was calculated as deaths x standard life expectancy at age of death.

GBD 2021 treats metabolic risk factors as quantifiable physiological states tied to NCDs and listed in the GBD 2021 taxonomy.^7^ For CKD attribution in this study, metabolic risk factors were restricted to high fasting plasma glucose (FPG), high systolic blood pressure (SBP), and high body mass index (BMI). Impaired kidney function was not treated as an upstream exposure for CKD to avoid circular attribution. Each factor has a theoretical minimum risk exposure level (TMREL); exposure distributions and relative risks are combined to compute population-attributable fractions (PAFs) and attributable DALYs. GBD’s four-tier system structures risks to avoid double-counting and support multilevel attribution: Level 1 = broad categories; Level 2 = clusters; Level 3 = modelable risks (e.g., high SBP, high FPG, high BMI); Level 4 = subcomponents of complex risks.^7^ The framework standardizes attribution, prevents double counting, and supports multi-level analyses.^4^,^7^

### Statistical analysis:

ASMR and ASDR, with 95% uncertainty intervals (UIs), were the main outcomes. Temporal trends were assessed using estimated annual percentage change (EAPC), with confidence intervals (CIs). Indicators were considered significantly increasing when both EAPC and its 95% CI were >0, decreasing when both were <0, and nonsignificant when the 95% CI included 0. Projections were generated using a Bayesian age-period-cohort (BAPC) model via the BAPC package in R, which accounts for nonlinear trends and cohort effects. Associations between SDI and ASMR/ASDR were analyzed with Pearson’s correlation, with significance set at P < 0.05. All statistical analyses and data visualizations were performed using R (version 4.4.2) and JD_GBDR (V2.37, Jingding Medical Technology Co., Ltd.).

## RESULTS

From 1990 to 2021, the global burden of CKD attributable to metabolic risk factors increased markedly. DALYs more than doubled, rising from 20,731,495.66 in 1990 to 44,438,400.64 in 2021; deaths nearly tripled during the same period, from 552,433.01 to 1,527,039.62 (Supplementary Table-SI & SII). The global ASDR rose from 479.67 per 100,000 in 1990 to 529.43 per 100,000 in 2021 (EAPC: 0.37). The ASMR increased from 14.85 per 100,000 to 18.49 per 100,000 (EAPC: 0.82), both indicating persistent upward trends. Sex-stratified analysis revealed that ASDR and ASMR were consistently higher in men than in women. Between 1990 and 2021, male ASDR increased from 546.20 to 603.16 per 100,000, while female ASDR rose from 426.76 to 465.55 per 100,000.

**Table-SI:** Comparison of deaths and ASMR for chronic kidney disease attributable to metabolic risk factors in 1990 and 2021, and estimated temporal changes during 1990-2021.

	1990		2021		1990–2021
	Death cases No. (95% UI)	ASMR (per 100, 000 population)	Death cases No. (95% UI)	ASMR (per 100, 000 population)	EAPC No. (95% CI)
Global	552433.01 (511666.12, 607550.67)	14.85 (13.62, 16.38)	1527039.62 (1382428.52, 1640064.35)	18.49 (16.63, 19.86)	0.82 (0.76, 0.89)
***Sex***					
Male	291535.78 (257066.75, 334565.58)	18.12 (16.12, 21.00)	794162.67 (715628.30, 855216.49)	21.90 (19.55, 23.57)	0.75 (0.69, 0.80)
Female	260897.23 (235194.14, 288241.82)	12.64 (11.44, 13.99)	732876.95 (652203.31, 796106.70)	15.89 (14.19, 17.26)	0.83 (0.75, 0.91)
***SDI***					
High SDI	99998.79 (91827.57, 104231.08)	9.22 (8.43, 9.62)	339997.06 (287768.86, 370305.00)	14.11 (12.25, 15.23)	1.73 (1.60, 1.86)
High-middle SDI	99170.25 (91252.97, 110707.06)	11.36 (10.41, 12.65)	226719.12 (200420.39, 252413.94)	12.02 (10.60, 13.38)	0.25 (0.16, 0.35)
Middle SDI	177238.32 (162246.81, 198217.43)	19.06 (17.31, 21.39)	512815.04 (457552.21, 556019.81)	20.88 (18.35, 22.66)	0.38 (0.28, 0.48)
Low-middle SDI	110835.42 (98300.89, 127753.14)	18.58 (16.35, 22.21)	309378.01 (280367.81, 349278.13)	23.07 (20.92, 26.33)	0.71 (0.66, 0.75)
Low SDI	64596.42 (57183.46, 73452.38)	29.69 (26.30, 34.58)	136729.73 (119064.07, 157236.72)	29.41 (26.04, 33.70)	-0.08 (-0.20, 0.04)
***GBD region***					
Andean Latin America	5793.05 (5259.59, 6475.09)	28.52 (25.86, 32.04)	21606.18 (17926.29, 25797.73)	37.64 (31.28, 44.96)	0.86 (0.52, 1.19)
Australasia	1851.11 (1680.24, 1960.98)	8.47 (7.62, 8.99)	5965.89 (4982.94, 6533.38)	9.63 (8.17, 10.49)	1.06 (0.81, 1.31)
Caribbean	4752.41 (4394.87, 5435.25)	18.73 (17.28, 21.29)	13882.37 (12025.12, 16174.33)	25.77 (22.32, 30.07)	1.48 (1.33, 1.63)
Central Asia	2535.11 (2248.62, 2930.07)	5.01 (4.38, 5.94)	9394.65 (8281.18, 10461.52)	12.11 (10.73, 13.43)	2.39 (1.89, 2.90)
Central Europe	14331.43 (13718.76, 14889.03)	10.47 (9.98, 10.93)	21630.30 (19430.79, 23978.39)	9.38 (8.40, 10.47)	-0.29 (-0.45, -0.13)
Central Latin America	22176.72 (21500.39, 22825.61)	27.93 (26.78, 28.86)	104403.57 (93847.09, 116541.59)	42.33 (38.11, 47.07)	1.81 (1.33, 2.30)
Central sub-Saharan Africa	8660.47 (7182.84, 10349.40)	42.65 (35.50, 50.59)	20998.75 (16149.59, 27060.51)	43.67 (33.29, 56.10)	-0.03 (-0.12, 0.07)
East Asia	107678.96 (94311.13, 126344.60)	14.37 (12.66, 16.99)	217227.36 (178377.61, 258421.53)	11.14 (9.16, 13.20)	-0.84 (-0.92, -0.75)
Eastern Europe	9387.44 (9170.96, 9572.47)	3.58 (3.50, 3.66)	17817.32 (16117.09, 19837.69)	5.22 (4.73, 5.81)	0.67 (0.31, 1.04)
Eastern sub-Saharan Africa	30585.49 (26859.46, 35142.28)	42.37 (37.03, 49.81)	60881.38 (53870.95, 69360.06)	40.07 (35.55, 45.97)	-0.36 (-0.44, -0.28)
High-income Asia Pacific	22227.54 (20127.74, 23363.60)	12.61 (11.21, 13.35)	63438.24 (50242.14, 71088.86)	9.74 (8.06, 10.69)	-0.92 (-1.00, -0.85)
High-income North America	29940.05 (27039.12, 31476.65)	8.32 (7.53, 8.74)	143649.42 (124290.61, 154905.77)	20.55 (18.00, 22.01)	3.40 (3.19, 3.61)
North Africa and Middle East	45450.43 (37637.71, 66170.44)	31.20 (25.45, 47.10)	144647.04 (126102.61, 163364.10)	37.70 (32.64, 42.46)	0.79 (0.63, 0.96)
Oceania	488.45 (342.77, 661.34)	17.21 (12.84, 23.27)	1545.01 (1266.95, 1878.79)	21.55 (17.95, 26.38)	0.64 (0.53, 0.76)
South Asia	79665.45 (68012.54, 89458.62)	13.98 (12.13, 15.91)	225926.04 (192147.16, 264583.10)	16.44 (13.97, 19.27)	0.42 (0.29, 0.54)
Southeast Asia	57847.23 (52067.59, 66478.80)	22.84 (20.53, 26.78)	169955.10 (148757.55, 190177.13)	28.46 (24.58, 31.84)	0.71 (0.66, 0.76)
Southern Latin America	11253.10 (10697.96, 11782.20)	26.00 (24.53, 27.24)	21576.19 (19408.20, 22965.97)	23.84 (21.52, 25.36)	-0.08 (-0.37, 0.22)
Southern sub-Saharan Africa	5372.29 (4671.46, 6593.98)	20.85 (17.98, 26.12)	17355.13 (15585.95, 19493.62)	34.42 (31.03, 38.33)	1.76 (1.38, 2.15)
Tropical Latin America	15519.42 (14835.74, 16084.35)	17.93 (16.80, 18.75)	46911.80 (42394.10, 49505.70)	18.84 (16.95, 19.91)	0.21 (0.05, 0.37)
Western Europe	48647.91 (44137.84, 50930.55)	8.26 (7.48, 8.66)	133441.65 (108749.55, 148525.80)	10.66 (8.84, 11.83)	1.35 (1.18, 1.52)
Western sub-Saharan Africa	28268.95 (24053.39, 32711.11)	33.62 (28.59, 39.12)	64786.24 (53465.93, 76089.36)	36.40 (31.08, 42.74)	0.20 (0.16, 0.24)

**Table-SII:** Comparison of DALYs and ASDR for chronic kidney disease attributable to metabolic risk factors in 1990 and 2021, and estimated temporal changes during 1990-2021

	1990		2021		1990–2021
	DALYs No. (95% UI)	ASDR (per 100,000 population)	DALY No. (95% UI)	ASDR (per 100,000 population)	EAPC No. (95% CI)
Global	20731495.66 (18818296.12, 22588237.70)	479.67 (438.38, 523.74)	44438400.64 (40799498.09, 48501692.54)	529.43 (484.78, 577.04)	0.37 (0.33, 0.41)
***Sex***					
Male	11064517.25 (9575324.72, 12298409.56)	546.20 (476.17, 612.42)	23750340.00 (21402819.35, 26128448.22)	603.16 (544.67, 661.91)	0.39 (0.36, 0.43)
Female	9666978.42 (8784636.45, 10589223.93)	426.76 (388.35, 467.47)	20688060.63 (18827240.06, 22721032.14)	465.55 (423.78, 511.32)	0.30 (0.25, 0.35)
***SDI***					
High SDI	2897983.21 (2603863.66, 3169731.91)	277.60 (249.89, 303.19)	7114609.99 (6455446.64, 7761279.88)	358.46 (324.68, 390.13)	1.07 (0.98, 1.16)
High-middle SDI	3533689.43 (3182601.74, 3956739.97)	360.77 (325.30, 401.56)	5942712.14 (5374281.41, 6630205.42)	324.55 (293.60, 361.15)	-0.35 (-0.42, -0.27)
Middle SDI	7029567.09 (6310878.67, 7801693.27)	585.38 (530.79, 653.23)	15694775.69 (14158611.19, 17139070.12)	596.22 (535.49, 650.29)	0.12 (0.03, 0.20)
Low-middle SDI	4687385.30 (4051784.11, 5165053.98)	609.15 (544.48, 685.62)	10607063.80 (9602029.38, 11765078.33)	686.72 (622.74, 765.77)	0.40 (0.38, 0.42)
Low SDI	2562236.58 (2262932.61, 2866139.00)	853.26 (759.30,969.65)	5039818.48 (4419578.17, 5816367.35)	791.46 (702.41, 906.49)	-0.33 (-0.42, -0.25)
***GBD region***					
Andean Latin America	190933.80 (174098.31, 211663.14)	753.75 (687.68, 839.91)	523956.59 (434691.85, 625866.59)	871.96 (723.54, 1038.58)	0.39 (0.11, 0.68)
Australasia	46400.40 (42169.79, 50537.81)	211.10 (192.22, 229.74)	113668.11 (101165.49, 125396.93)	216.57 (193.18, 239.21)	0.53 (0.35, 0.71)
Caribbean	161541.25 (149270.82, 180855.37)	564.93 (523.46, 635.35)	385143.88 (332007.73, 454087.91)	735.58 (632.79, 868.33)	1.25 (1.13, 1.37)
Central Asia	176272.17 (152266.06, 202028.31)	326.05 (278.34, 378.86)	427569.01 (377188.89, 490078.85)	493.12 (435.38, 564.27)	1.02 (0.67, 1.37)
Central Europe	469571.38 (436082.04, 504778.10)	342.73 (318.20, 368.37)	539889.77 (472336.55, 611913.91)	266.87 (233.19, 305.87)	-0.74 (-0.84, -0.63)
Central Latin America	779710.05 (736746.82, 822266.35)	767.71 (723.82, 811.55)	2992672.92 (2691685.99, 3368404.74)	1170.73 (1055.67, 1314.98)	1.75 (1.31, 2.20)
Central sub-Saharan Africa	349698.20 (292472.36, 409340.07)	1157.95 (981.26, 1362.51)	783235.42 (623378.18, 989801.28)	1124.15 (901.15, 1425.41)	-0.19 (-0.26, -0.12)
East Asia	4371824.43 (3838739.78, 5004508.84)	461.19 (405.95, 530.01)	6483531.84 (5529079.18, 7585877.12)	322.22 (275.68, 377.19)	-1.18 (-1.27, -1.09)
Eastern Europe	521645.89 (463276.76, 581678.29)	207.40 (184.64, 230.84)	642388.74 (562736.58, 726824.73)	204.65 (180.31, 232.19)	-0.58 (-0.78, -0.37)
Eastern sub-Saharan Africa	1116542.74 (971175.07, 1249761.72)	1094.26 (961.86, 1254.65)	2046284.61 (1799105.13, 2367216.48)	947.83 (839.32, 1087.85)	-0.65 (-0.74, -0.57)
High-income Asia Pacific	599486.75 (547911.41, 643910.75)	315.61 (288.83, 339.35)	1146352.81 (983028.41, 1269635.76)	235.52 (204.66, 259.79)	-0.92 (-0.98, -0.87)
High-income North America	894944.81 (797846.03, 986189.36)	266.59 (237.70, 293.11)	3120540.29 (2863651.16, 3352222.94)	508.74 (467.44, 545.63)	2.45 (2.30, 2.61)
North Africa and Middle East	1492778.08 (1271571.19, 1956657.51)	759.58 (642.77, 1055.25)	3925118.39 (3427544.71, 4431804.58)	846.43 (747.64, 947.99)	0.51 (0.40, 0.61)
Oceania	22657.60 (16724.37, 29075.90)	583.27 (445.39, 739.74)	65794.24 (55701.55, 77161.80)	698.85 (598.04, 818.69)	0.52 (0.42, 0.62)
South Asia	3672373.77 (3151758.37, 4111353.02)	509.54 (447.27, 572.70)	8439808.65 (7355624.67, 9697877.78)	540.35 (473.06, 620.46)	0.13 (0.08, 0.18)
Southeast Asia	2407426.06 (2132563.57, 2693072.61)	751.57 (668.34, 850.96)	5701052.30 (5025491.44, 6329437.10)	845.93 (749.10, 940.17)	0.38 (0.35, 0.41)
Southern Latin America	281039.64 (266500.90, 294509.37)	612.60 (580.76, 641.75)	441029.16 (410797.90, 468321.58)	515.09 (481.14, 546.68)	-0.37 (-0.60, -0.14)
Southern sub-Saharan Africa	202852.14 (179709.50, 234037.62)	623.48 (548.00, 739.79)	551179.13 (492905.79, 619550.40)	895.52 (805.01, 997.81)	1.32 (0.97, 1.67)
Tropical Latin America	594768.52 (559113.86, 631144.26)	562.60 (527.64, 598.17)	1311621.32 (1217241.64, 1401052.64)	516.86 (479.46, 551.84)	-0.31 (-0.44, -0.18)
Western Europe	1267362.84 (1122802.22, 1409718.16)	234.08 (206.64, 260.10)	2360882.39 (2067887.80, 2642390.19)	241.68 (209.79, 271.31)	0.38 (0.28, 0.47)
Western sub-Saharan Africa	1111665.10 (950727.54, 1277816.31)	928.00 (798.58, 1065.33)	2436681.08 (1987644.81, 2885885.66)	930.64 (786.86, 1081.61)	-0.04 (-0.08, 0.01)

**Fig.1:**
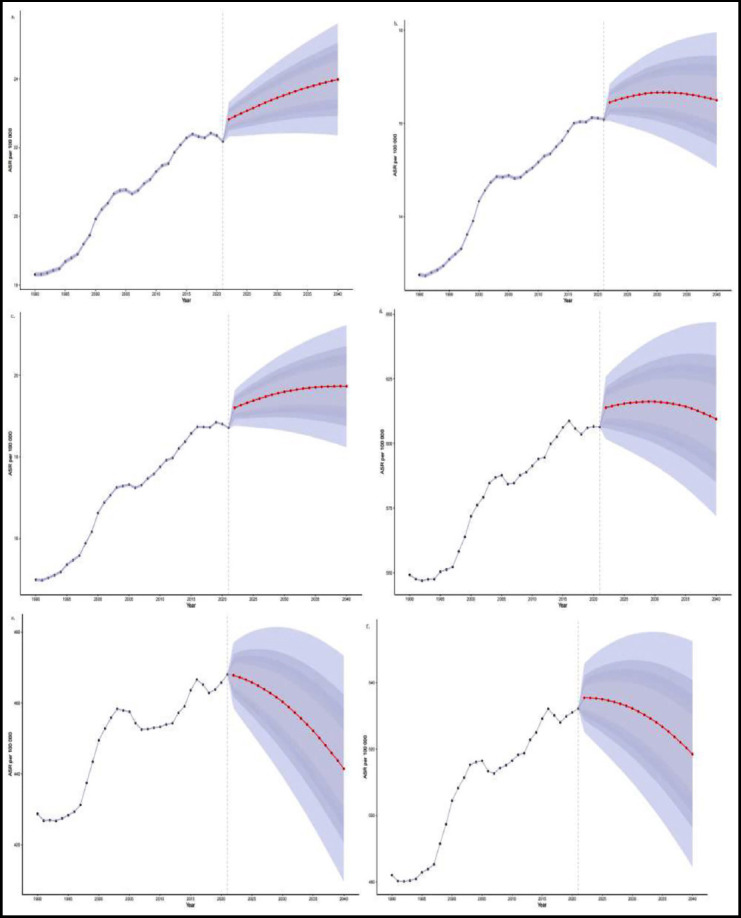
Projected age-standardized mortality rate (ASMR) and age-standardized disability-adjusted life-years rate (ASDR) for chronic kidney disease (CKD) attributable to metabolic risk factors from 1990 to 2040. Panels: (A) male ASMR; (B) female ASMR; (C) global ASMR; (D) male ASDR; (E) female ASDR; (F) global ASDR. Shaded areas indicate 95% uncertainty intervals.

Projection analysis (Fig.1) indicated that global ASMR is projected to continue a slow rise, reaching 19.73 per 100,000 by 2040. Male ASMR is expected to increase from 21.90 (2021) to 23.98 per 100,000 (2040); female ASMR is projected to peak around 2031 (16.66 per 100,000) before slightly declining to 16.50 per 100,000 by 2040. Conversely, global ASDR is projected to follow a downward trend, falling to 518.47 per 100,000 by 2040, with this trend being more pronounced in women.

**Supplementary Fig.S1:**
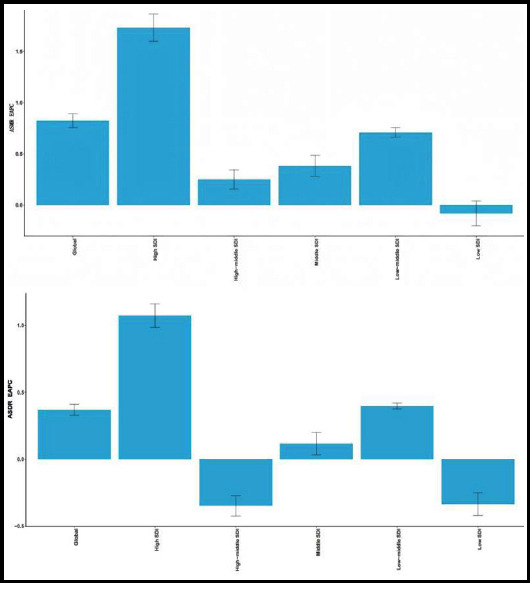
Estimated annual percentage change (EAPC) of ASMR and ASDR for CKD attributable to metabolic risk factors across the five SDI regions, 1990-2021. CKD, chronic kidney disease; ASMR, age-standardized mortality rate; ASDR, age-standardized DALY rate; SDI, Socio-demographic Index.

In 2021, the Middle SDI region reported the highest absolute number of CKD deaths (512,815.04). However, the Low-SDI region recorded the highest ASMR (29.41 per 100,000) and ASDR (791.46 per 100,000) globally (Supplementary Table-SI & SII, Fig.S1). The High-SDI region had the lowest ASMR (14.11 per 100,000), while the High-middle SDI region had the lowest ASDR (324.55 per 100,000).

**Supplementary Fig.S2:**
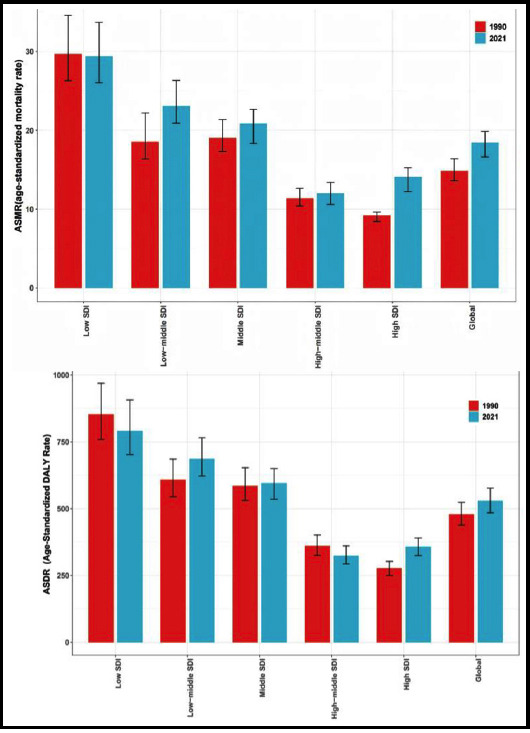
ASMR and ASDR of CKD attributable to metabolic risk factors across the five SDI regions in 1990 and 2021. CKD, chronic kidney disease; ASMR, age-standardized mortality rate; ASDR, age-standardized DALY rate; SDI, Socio-demographic Index.

Between 1990 and 2021 (Supplementary Fig.S2), the High-SDI region experienced the largest increases in both ASMR (EAPC: 1.73) and ASDR (EAPC: 1.07). Conversely, in the Low-SDI region, ASDR declined, whereas ASMR remained broadly stable.

**Supplementary Fig.S3:**
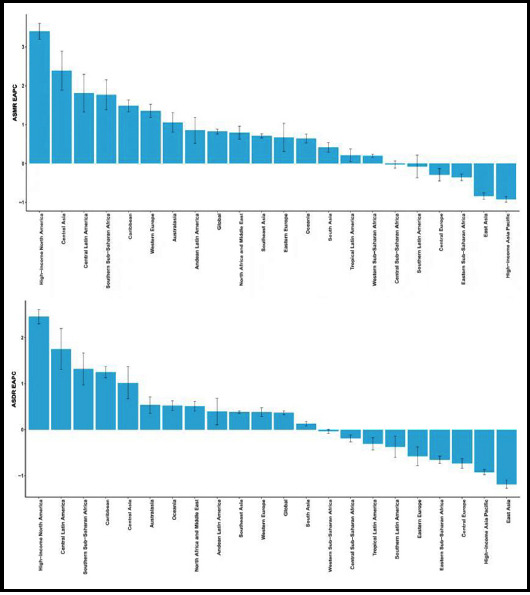
Estimated annual percentage change (EAPC) of ASMR and ASDR for CKD attributable to metabolic risk factors across 21 GBD regions, 1990-2021. CKD, chronic kidney disease; ASMR, age-standardized mortality rate; ASDR, age-standardized DALY rate; GBD, Global Burden of Disease.

Geographically (Supplementary Fig.S3), South Asia reported the highest absolute number of deaths (225,926.04) and DALYs (8,439,808.65) in 2021. However, the highest standardized rates were observed in Central Sub-Saharan Africa for ASMR (43.67 per 100,000) and Central Latin America for ASDR (1,170.73 per 100,000).

**Supplementary Fig.S4:**
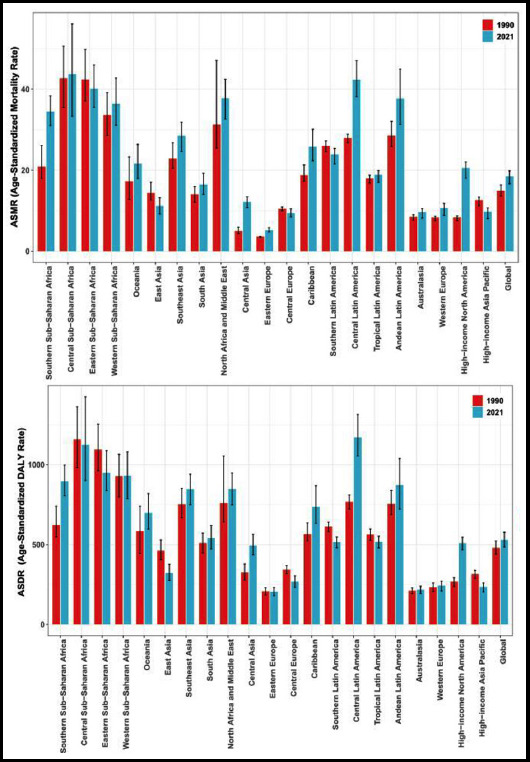
ASMR and ASDR of CKD attributable to metabolic risk factors across 21 GBD regions in 1990 and 2021. CKD, chronic kidney disease; ASMR, age-standardized mortality rate; ASDR, age-standardized DALY rate; GBD, Global Burden of Disease.

From 1990 to 2021 (Supplementary Fig.S4), High-income North America showed the largest increases in ASMR (EAPC: 3.40) and ASDR (EAPC: 2.45). In contrast, regions including Central Europe, East Asia, and parts of Sub-Saharan Africa demonstrated declining trends.

**Fig.2:**
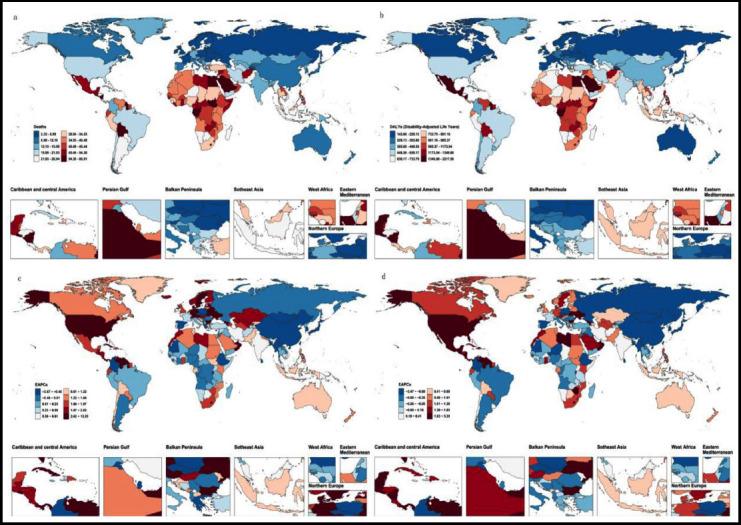
Global distribution and temporal trends of CKD attributable to metabolic risk factors in 204 countries and territories. (A) ASMR in 2021; (B) ASDR in 2021; (C) estimated annual percentage change (EAPC) in ASMR from 1990 to 2021; (D) EAPC in ASDR from 1990 to 2021. CKD, chronic kidney disease; ASMR, age-standardized mortality rate; ASDR, age-standardized DALY rate; DALY, disability-adjusted life-year; EAPC, estimated annual percentage change.

At the national level in 2021 (Fig.2), China (204,118.09), India (175,540.67), and the United States (135,852.95) had the highest number of attributable deaths. Mauritius reported the world’s highest ASMR (80.11 per 100,000) and ASDR (2,195.63 per 100,000).

**Fig.3:**
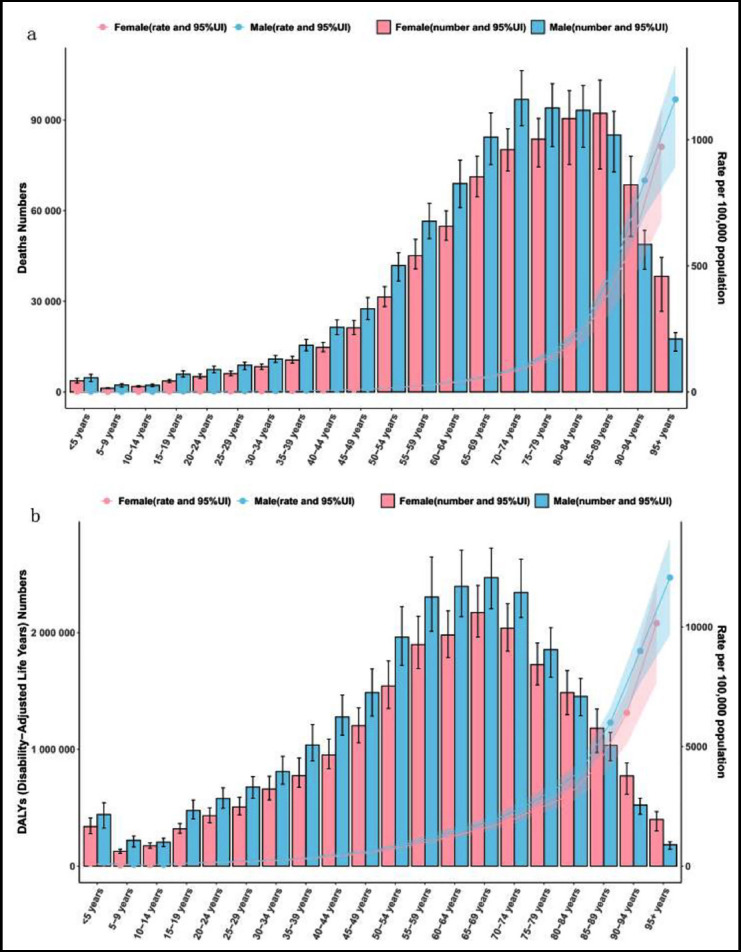
Age- and sex-specific CKD burden attributable to metabolic risk factors in 2021. (A) Deaths and age-specific mortality rate; (B) DALYs and age-specific DALY rate. CKD, chronic kidney disease; DALY, disability-adjusted life-year.

Age-specific analysis for 2021 (Fig.3) showed that age-specific mortality and DALY rates increased with age. Male age-specific mortality rates were higher than female rates across most age groups. Deaths peaked at ages 70-74 years in males and 85-89 years in females, while DALYs peaked at ages 65-69 years for both sexes. A visible early-life elevation was also observed among children under five, with a higher mortality-related burden than in adjacent pediatric age groups.

**Fig.4:**
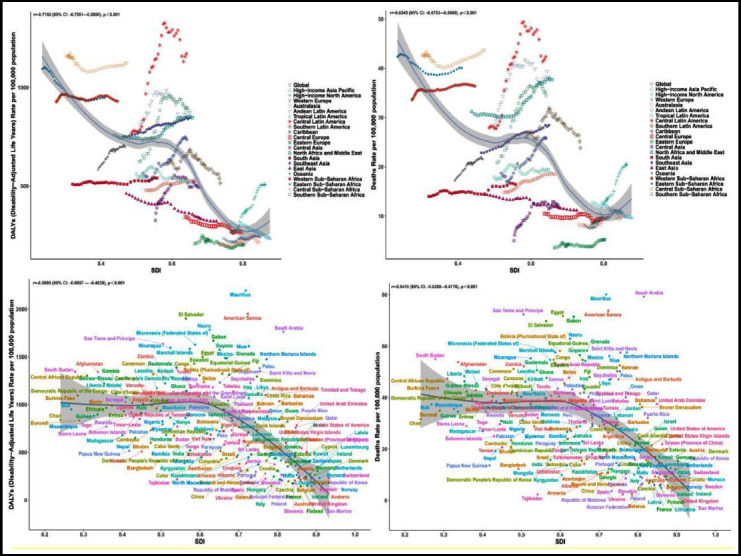
Correlation between Socio-demographic Index (SDI) and age-standardized CKD burden attributable to metabolic risk factors in 2021. (A) Regional ASDR versus SDI (n = 21); (B) regional ASMR versus SDI (n = 21); (C) country-level ASDR versus SDI (n = 204); (D) country-level ASMR versus SDI (n = 204). CKD, chronic kidney disease; ASMR, age-standardized mortality rate; ASDR, age-standardized DALY rate; SDI, Socio-demographic Index.

**Supplementary Fig.S5:**
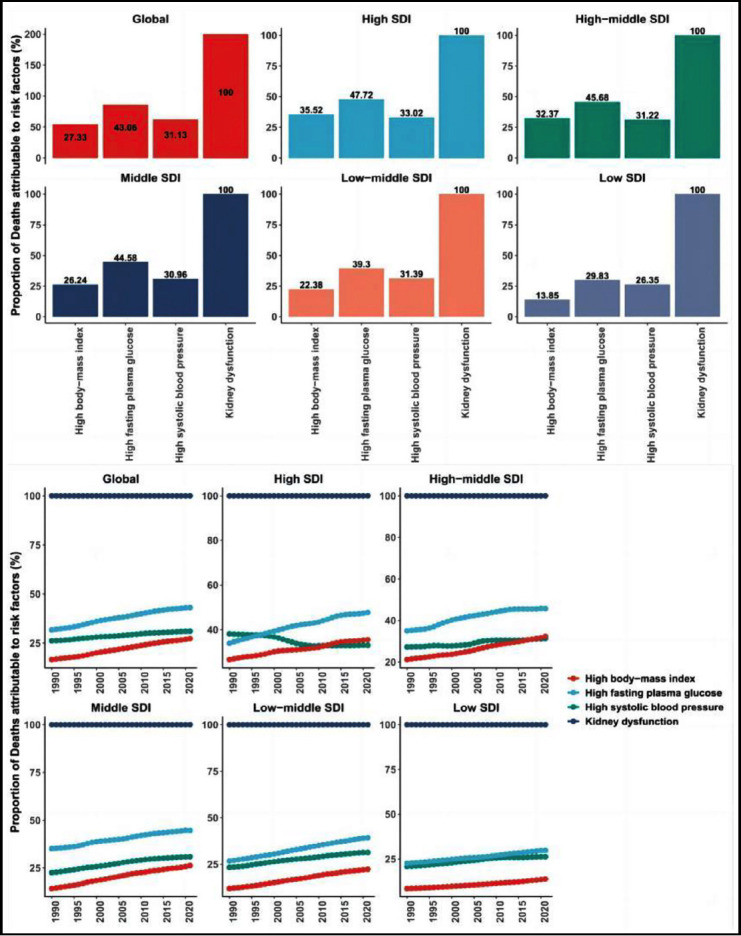
Contribution of high fasting plasma glucose (FPG), high systolic blood pressure (SBP), and high body mass index (BMI) to CKD mortality by SDI region and their temporal trends from 1990 to 2021.

CKD, chronic kidney disease; SDI, Socio-demographic Index.

In the SDI correlation analysis (Fig.4), both ASMR (r = -0.541, p < 0.001) and ASDR (r = -0.599, p < 0.001) showed significant overall inverse correlations with SDI, suggesting that countries with higher SDI generally had a lower age-standardized burden of CKD attributable to metabolic risk factors. Globally in 2021 (Supplementary Fig.S5), high fasting plasma glucose (43.06%) was the leading metabolic risk factor for mortality, followed by high systolic blood pressure (31.13%) and high BMI (27.33%). This ranking was consistent across most SDI regions. In High-SDI regions, however, the mortality proportion from high BMI (35.52%) surpassed that from high systolic blood pressure (33.02%). The attributable proportion of high BMI declined from High-SDI to Low-SDI regions, from 35.52% to 13.85%.

## DISCUSSION

Using GBD 2021 data, this study found that the burden of CKD attributable to metabolic risk factors increased substantially from 1990 to 2021. Deaths rose faster than the age-standardized DALY rate (ASDR), the burden remained higher in men and older adults, and marked heterogeneity was observed across SDI levels and regions. Compared with previous GBD studies that mainly described the overall CKD burden, this analysis focused on the metabolic-risk-attributable component and further separated the contributions of high fasting plasma glucose (FPG), high systolic blood pressure (SBP), and high body mass index (BMI) across SDI strata.^3^,^4^,^7^ These findings are consistent with integrated GBD analyses showing that metabolic risk accumulation has become an increasingly important contributor to non-communicable disease burden in younger and middle-aged populations.^11^

The observed upward trend is broadly consistent with recent GBD 2021 analyses, which identified high FPG, high SBP, and high BMI as major drivers of CKD burden.^4^,^7^ Studies of type 2 diabetes-related CKD also indicate that population growth, ageing, and increasing exposure to hyperglycemia largely explain the long-term increase in disease burden.^12^ However, our findings further show that the burden is not evenly distributed: low-SDI regions had the highest age-standardized mortality rate (ASMR) and ASDR, whereas high-SDI regions showed the fastest increases, particularly in BMI-related burden. This extends previous work by suggesting that metabolic risk contributions should be interpreted in relation to development level rather than as a uniform global exposure pattern.

We found significant overall inverse correlations between ASMR/ASDR and SDI. Mortality and DALY rates generally declined with socioeconomic development, although the increasing contribution of high BMI in high-SDI settings may reflect the combined effects of obesity, sedentary behavior, energy-dense diets, and prolonged survival among people with diabetes who remain at risk of CKD progression.^1^,^3^ This pattern agrees with the Global Kidney Health Initiative view that CKD is a multifactorial condition shaped by socioeconomic transition, health-system capacity, and lifestyle change.^13^ Demographic change also contributed: population growth and ageing increased the absolute numbers of CKD cases and DALYs,^14^ while earlier and longer exposure to high FPG and high BMI may partly explain the faster rise in age-standardized rates in middle- and high-SDI regions.^15^ In contrast, delayed diagnosis, insufficient diabetes and hypertension control, and limited access to kidney care may sustain high DALYs and mortality in low- and low-middle-SDI settings.^13^

The age-sex pattern has practical implications. Men had higher ASMR and ASDR, with a mortality peak at 70-74 years, whereas deaths among women exceeded those among men at very old ages, consistent with previous reports of sex differences in CKD burden.^16^ The higher male burden may relate to cardiovascular comorbidity, smoking, occupational exposures, and poorer control of metabolic disease, while the excess burden in older women may reflect longer survival, postmenopausal metabolic changes, and frailty-related vulnerability.^5^,^17^ The disproportionately elevated mortality signal in children under five should be interpreted cautiously. It may reflect congenital or hereditary kidney disease, severe early-life metabolic or cardiovascular complications, underlying-cause misclassification, or wider uncertainty in sparse pediatric data. Because GBD attribution cannot distinguish these mechanisms, this finding should be regarded as hypothesis-generating rather than causal.

Risk-factor decomposition clarifies prevention priorities. Stratified analyses indicated that high FPG, high SBP, and high BMI were the leading metabolic drivers of CKD deaths and DALYs; cohort, trial, and mechanistic evidence broadly supports this profile.^18^,^19^ For high FPG, cohort studies and reviews show a dose-response relationship between impaired glucose metabolism and CKD progression; early detection and glycemic control can reduce albuminuria and slow GFR loss.^17^,^20^ High SBP remains a key determinant of CKD progression; guideline-based blood pressure control, renin-angiotensin system blockade, diuretics when appropriate, and sodium-glucose cotransporter-2 inhibitors can reduce renal and cardiovascular risk in eligible patients.^21^–^23^ High BMI contributes indirectly through diabetes and hypertension and directly through hyperfiltration, lipotoxicity, chronic inflammation, and hemodynamic stress.^13^,^24^ Therefore, the rising BMI-attributable CKD burden in high- and upper-middle-SDI regions should be a major policy concern. High sodium intake may further amplify CKD burden through blood pressure and metabolic pathways.^25^

SDI-stratified risk patterns further support targeted rather than uniform interventions. In upper-middle- and high-SDI regions, the faster rise in BMI-attributable CKD is consistent with lifestyle transition and obesity expansion.^26^ In low- and low-middle-SDI regions, delayed diagnosis, inadequate treatment, and poor complication management remain important reasons for the high per-capita burden.^27^ H-CKD reports indicate only modest ASMR gains in resource-limited settings, reflecting gaps in primary care and kidney services,^28^ while low physical activity has also become a growing CKD-related burden in low-SDI settings.^29^ Thus, prevention should match the dominant risk profile of each setting. Low- and low-middle-SDI regions should prioritize low-cost screening for albuminuria, serum creatinine, blood pressure, and diabetes; standardized management of hypertension and hyperglycemia in primary care; access to essential medicines; salt and sugar reduction; and timely referral of high-risk patients.

Middle-SDI regions need integrated diabetes-hypertension-CKD management and risk-based follow-up. High- and upper-middle-SDI regions should prioritize obesity prevention and control through dietary policy, physical-activity promotion, structured weight management, and, where appropriate, anti-obesity drugs and kidney-protective therapies such as SGLT2 inhibitors and GLP-1 receptor agonists.^13^,^22^,^23^,^30^ Globally, CKD should be more fully incorporated into non-communicable disease agendas, with strengthened renal replacement therapy capacity, multidisciplinary care, and linkage with cardiovascular, diabetes, and obesity programs according to local resources.^21^

### Strength

The major strength of this study is that it used the standardized GBD 2021 framework to evaluate the attributable pathway from metabolic risk factors to CKD outcomes across age, sex, SDI, region, and country.^7^ Unlike single-risk analyses, this approach better reflects the multimorbidity context of CKD and identifies how risk-factor contributions differ by development level. The combination of historical trend assessment and BAPC projections also helps distinguish areas with the highest current burden from those likely to experience the fastest future increase.

### Limitations

First, GBD estimates depend on available data, model assumptions, and covariates; measurement error and residual misclassification are therefore possible, especially in low-SDI regions with incomplete surveillance. Second, uncertainty may be greater for children, small countries, and settings with limited laboratory or death-registration data, so regional comparisons and findings in children under five should be interpreted cautiously. Third, attributable estimates are based on population-level exposure and relative-risk inputs and cannot prove individual-level causality. Finally, although trials and guidelines support kidney-protective therapies such as SGLT2 inhibitors, real-world effectiveness depends on affordability, adherence, health-system coverage, and timely diagnosis, which were beyond the scope of this analysis. Future studies should validate high-risk signals using primary datasets and assess implementation strategies suited to different resource settings.

## CONCLUSIONS

Using GBD 2021, this study showed that CKD attributable to metabolic risk factors increased markedly from 1990 to 2021, with shifting risk-factor contributions and an overall inverse association with SDI; high fasting plasma glucose, high systolic blood pressure, and high body mass index remain priority targets. Response strategies should be SDI-, age- and sex-specific, prioritizing obesity control and sodium-glucose cotransporter-2 inhibitors for eligible patients in high-SDI regions, and early screening plus diabetes and hypertension management in low-SDI regions.

### Authors’ Contributions:

**YY** and **QQ:** responsible for conceptualization, methodology design, and formal analysis.

**XZ**, **PL:** contributed to investigation, software development, data curation, visualization, and manuscript drafting.

**LZ:** supervised the work and contributed to critical revision of the manuscript.

All authors participated in the interpretation of findings, reviewed the manuscript critically for intellectual content, and approved the final version for submission.
